# Randomised controlled feasibility study protocol of the Carers-ID online intervention to support the mental health of family carers of people with intellectual disabilities

**DOI:** 10.1186/s40814-024-01448-w

**Published:** 2024-02-06

**Authors:** Mark A. Linden, Rachel Leonard, Trisha Forbes, Michael Brown, Lynne Marsh, Stuart Todd, Nathan Hughes, Maria Truesdale

**Affiliations:** 1 Medical Biology Centre, School of Nursing and Midwifery, Queen’s University, 97 Lisburn Road, Belfast, BT9 7BL Ireland; 2 School of Care Sciences, University of South Wales, Caerleon, Wales; 3 Department of Sociological Studies, University of Sheffield, Sheffield, England; 4 School of Health and Wellbeing, University of Glasgow, Glasgow, Scotland

**Keywords:** Family carers, Feasibility, Randomised controlled trial, Mental health, COVID-19, Intellectual disability, Protocol

## Abstract

**Background:**

Family carers play a crucial role in supporting the health and well-being of people with intellectual disabilities. Given their role and responsibilities, many family carers experience significant and ongoing stress and mental health difficulties. Programmes and interventions which provide training and support to family carers have been shown to have a positive impact on levels of stress and quality of life. However, these are often face to face which can create barriers to full participation. Online interventions have been shown to offer flexibility in delivery compared with traditional face-to-face approaches. The primary objective of this study is to determine the feasibility of delivering the Carers-ID online intervention, while the secondary outcome is improved mental health in family carers of people with intellectual disabilities.

**Methods:**

Family carers (*n* = 120) will be randomised to receive the intervention (*n* = 60) or assigned to a wait-list control (*n* = 60) group. The intervention (www.Carers-ID.com) consists of 14 modules which cover topics including the following: promoting resilience, providing peer support, reducing anxiety, managing stress, accessing local supports and managing family conflict and information for siblings who are carers. The intervention has been co-produced with voluntary sector organisations and family carers and tested for acceptability. Primary outcomes for this study include acceptability and feasibility of the outcome measures, recruitment, participation and retention rates and effect sizes. Secondary outcomes will be completed at three time points (baseline, following intervention completion and 3 months after completion). These include the following: the Depression, Anxiety and Stress Scale, the Warwick–Edinburgh Mental Well-being Scale, the Resilience Scale and the Social Connectedness Scale Revised. Participants (*n* = 12) who have taken part in the intervention arm of the research will be invited to participate in semi-structured interviews as part of the process evaluation.

**Discussion:**

The Carers-ID intervention provides an online resource for family carers to support their mental health and well-being and promote their resilience. It represents an affordable and accessible means of delivering such support. Testing the feasibility of the intervention and related trial procedures is required to determine whether a full-scale randomised controlled trial to evaluate the intervention’s effectiveness is warranted.

**Trial registration:**

ClinicalTrials.gov: NCT05737823

## Background

The majority of people with intellectual disabilities are living at home with their family, and family carers are providing ongoing and multifaceted care for their family members across the lifespan. Care can include any or all of the following: medication management, monitoring a physical condition, direct care, arranging and managing community presence and participation, liaising with professionals, supportive interventions, and behaviour management [1]. As approximately 77% of people with ID in England lived with their families in 2017–2018 [2], family carers play a crucial role in supporting their health and well-being. However, many family carers experience significant and ongoing stress and mental health difficulties [3, 4]; many also experience the positive aspects of caring for a family member who has ID [5, 6].

Programmes and interventions which provide training and support for family carers have been shown to have a positive impact on the following: levels of stress and feelings of confidence [7], child socialisation [8], and quality of life [9]. However, some family carers may face barriers in taking part in such interventions including time, family pressures, cost, and availability of services [10]. Online programmes offer greater flexibility of delivery and have been shown to offer comparable outcomes to more traditional treatment approaches in areas such as parenting styles [11], knowledge, and self-efficacy [12]. However, while online programmes show tangible benefits for family carers, few have been designed in collaboration with carers to better address their needs (Forbes T, Brown M, Marsh L, Truesdale M, McCann E, Todd S, et al: Online support programmes for family carers of people with intellectual disabilities: systematic review of the international evidence base, under review).

The COVID-19 pandemic made access to face-to-face care and support even more challenging for families, with some services offering online alternatives [13, 14]. While this created challenges for many, including access to reliable technology, it has also provided opportunities, such as reduced travel and delivery costs, and improved access to programmes [15].

A previous study conducted in 2021 by the same authors explored the impact of the COVID-19 pandemic on family carers. Based on these findings, a programme to support family carers of people with Profound and Multiple Intellectual Disabilities was developed — www.Carers-ID.com. The programme, co-produced with family carers and voluntary sector organisations across the UK and Ireland, sought to provide connectedness, reliable and easily accessible evidence-based materials and information on how to support mental health.

### Aim and objectives

The current study will be conducted in line with the updated Medical Research Council (MRC) framework for the development of complex interventions [16]. According to the framework, testing of intervention procedures, establishing recruitment and retention rates and determining a suitable sample size are key elements in the development of complex interventions. Therefore, the primary aim of this study is to determine the feasibility of conducting a large-scale effectiveness trial of the Carers-ID intervention. Specifically, we will determine the following:

1. Recruitment, retention and attrition rates of participation in the trial.
2. Intervention engagement and adherence
3. Potential effect sizes and variability to inform a sample size calculation in an effectiveness trial
4. Feasibility of collecting outcome data using measures of stress, anxiety, depression, resilience and well-being in assessing the impact of the intervention on family carers

The secondary aims are as follows:

1. Examine impact on family carers mental health outcomes (stress, anxiety, depression, well-being and resilience) to guide a future trial.

## Methods

### Design

This is a randomised controlled feasibility design utilising a wait-list control with data collection at baseline (T0), on intervention completion, 2 weeks after baseline (T1) and at 3 months from baseline (T2). It will employ a qualitative process evaluation to explore acceptability of the outcome measures [17]. This trial is registered with the US National Library of Medicine, Clinical Trials Register (ID: NCT05737823), and will be reported in accordance with the Consolidated Standards of Reporting Trials (CONSORT) statement for pilot and feasibility studies [18]. Table 1 describes the flow of participants through the trial and the schedule of intervention and assessment.

**Table 1:** Schedule of intervention and assessment

	Intervention group	Control group
**Time 1 — baseline (T0)**	Baseline measures	Baseline measures
**2 weeks**	Complete intervention	No active treatment
**Time 2 — 2 weeks after baseline (T1)**	2nd measures	2nd measures
**Interviews**	Acceptability interviews	No interview completed
**Time 3 — 3 months from baseline (T2)**	3rd measures	3rd measures
**Access to intervention**	-	Gain access to intervention

### Participants and recruitment

Based on previous studies [19, 20] and best practice guidelines [19, 21] we estimate, we will need to recruit between 100 and 120 family carers who will be randomised to receive the intervention (*n* = 60) or assigned to a wait-list control (*n* = 60) group. The sample characteristics will be similar to the target population of the future large-scale effectiveness trial. Participants will be UK adults > 18 years of age and will be caring for a family member with an ID (people with all levels of ID). As this is a feasibility trial, the sample size is not intended to detect a statistically significant difference. However, these numbers will be sufficient to allow us to test the trial methodology and acceptability and feasibility of our psychometric measures [22, 23]. Data gained from this study will allow us to calculate a sample size for a future effectiveness trial.

Participants will be recruited through our voluntary sector partners. Letters of invitation and information sheets will be sent out to members of our partner organisations to inform them of the study trial. Those interested in taking part will contact the research team if they require further information or to indicate their wish to participate.

For the process evaluation, participants (*n* = 12) who have taken part in the intervention arm of the study will be invited to participate in an online interview. An invitation letter and information sheet will be sent to all participants in the intervention group by the researcher. Those interested in taking part will contact the research team if they require further information or to indicate their wish to participate.

### The intervention

The intervention (www.Carers-ID.com) is delivered online and comprises video and audio accounts of family carers’ experiences, peer-to-peer support, resources and activities which promote resilience and improve mental health. Carers-ID consists of 14 modules which cover topics including the following: promoting resilience, providing peer support, reducing anxiety, managing stress, accessing local supports and managing family conflict and information for siblings who are carers. It also shares examples of carers’ experiences of the COVID-19 pandemic and offers day-to-day accounts of successful strategies individuals used to improve their mental health. Our research showed that family carers wanted to feel connected to others and enjoyed sharing their experiences, and the Carers-ID intervention connects family carers with each other so they can provide peer support.

### Outcomes and success criteria

#### Primary outcomes

Criteria to assess the feasibility of progressing to a large-scale effectiveness trial include the following:

1. Acceptability and feasibility of the outcome measures (completed by > 80% of family carers)
2. Sufficient recruitment (> 90 carers) and participation and retention rates (> 80% of family carers)
3. Effect sizes and estimate of variability (standard deviation), along with data from previous studies, may inform a sample size for a future effectiveness trial.

The primary outcome of this trial is to determine the feasibility of conducting a future effectiveness trial of the Carers-ID intervention, including acceptability and feasibility of the outcome measures, recruitment, participation and retention rates and effect sizes and estimates of variability.

Data on numbers of participants identified, recruited, commenced and finished in the intervention will be collected throughout the study. Reasons for declining participation and reasons for drop out will be recorded where possible. Feasibility of using the stress, anxiety, depression, resilience and well-being outcomes will be assessed through number of participants completing pre, post and follow-up measures. Acceptability of measures will be assessed qualitatively through semi-structured interviews (see below). We will also collect data on participant’s activity within the online intervention, including total time spent on the intervention and number of modules accessed, to enable us to assess intervention engagement and adherence.

#### Secondary outcomes measures

Our psychometrically validated outcome measures comprise the Depression, Anxiety and Stress Scale-21 [24], the Warwick–Edinburgh Mental Well-being Scale [25], the Resilience Scale [26] and the Social Connectedness Scale Revised [27]. Participant demographics such as age, sex, country (England, Scotland, Wales and Northern Ireland) and relationship to person with ID will also be collected.

The Depression, Anxiety and Stress Scale-21 items (DASS-21) [24] are a set of three self-report sub-scales designed to measure the emotional states of depression, anxiety and stress. Each of the three DASS-21 sub-scales contains 7 items, with 21 items in total. Items are rated on a 4-point Likert scale, from ‘Did not apply to me at all’ to ‘Applied to me very much or most of the time’. Scores for depression, anxiety and stress are calculated by summing the scores for the relevant items and multiplying by 2, with potential scores ranging from 0 to a maximum of 42, as scoring is based on the full 42-item version [24]. The scale has excellent reliability, with Cronbach’s alphas of 0.94, 0.88 and 0.93 for depression anxiety and stress respectively [24].

The Warwick-Edinburgh Mental Well-being Scale (WEMWBS) was developed to enable the measurement of mental well-being and has excellent reliability with a Cronbach’s alpha of 0.91 [25]. The WEMWBS is a 14-item scale of positively worded statements covering feeling and functioning aspects of mental well-being. The 14 statements have five response categories from ‘none of the time’ to ‘all of the time’. The WEMWBS is scored by summing the scores for each of the 14 items, which are scored from 1 to 5. Scores range from 14 to 70 with higher scores indicate greater positive mental well-being [25]. Previous research found that a change of about 3 or more points can be considered clinically significant [26].

The 25-item Resilience Scale was developed as a general measure of resilience for adults across the lifespan [26]. Participants respond by either agreeing or disagreeing with statements on a scale of 1 (disagree) to 7 (agree). The responses are summed (minimum score of 25 to a maximum of 175) with higher score reflecting stronger resilience. Reported Cronbach’s alpha for this scale range from 0.87 to 0.95 [27].

The 20-item Social Connectedness Scale Revised (SCS-R) is used to assess the extent to which persons feel connected to others in their surrounding social area [28]. Responses are on a Likert scale with 1 ‘strongly disagree’ to 6 ‘strongly agree’. Negatively worded items are reverse scored and summed together with the positively worded items to create a score ranging from 20 to 120. Higher scores on the SCS-R reflect a stronger sense of social connectedness. The scale has a Cronbach’s alpha of 0.92 [28].

### Process evaluation

Feasibility and acceptability of our outcome measures will also be determined by gaining the perspectives of family carers who took part in the intervention arm. Semi-structured interviews will be conducted post intervention (see Table 1) and held via a secured online platform, and interviews will be recorded, transcribed verbatim, and subjected to thematic analysis [29]. The interview schedule was developed to explore ways to make improvements to the Carers-ID intervention. Questions focused on the overall experience, relevance, usefulness, and potential impact of the intervention. For example, ‘What improvements do you think could be made to the Carers-ID programme?’ and ‘What has been the impact of the Carers-ID online programme on you and/or your family?’

### Procedures

Ethical approval has been granted by a university ethics committee at the lead authors institution (MHLS 23_04). Participants will be required to give informed consent before commencing the study. Participants will be asked to complete the psychometrically validated questionnaires at three time points: prior to taking part in the intervention, baseline measurement (T_0_), 2 weeks following baseline/completion of intervention (T_1_), and 3 months after baseline(T_2_). Semi-structured interviews will be conducted, as part of the process evaluation, with 12 participants to determine acceptability and feasibility of the outcome measures and the intervention.

Random allocation to the intervention and control conditions will be undertaken by an independent third party not involved with the research. The allocation ratio used will be 1:1. Random sequences will be generated using the Random.org (https://www.random.org/lists/) online service which uses atmospheric noise to ensure randomness. This will ensure members of the research team will be blind to allocation to reduce selection bias. Participants will complete the psychometric measures online with no data collection by members of the research team. Blinding of the research team to outcome assessment should have the effect of reducing detection bias. All participants will receive the intervention with those in the control group receiving this at a later stage. Due to the difference in timings, participants may become aware of group allocation.

### Data analysis

All analysis will be conducted on an ‘intention-to-treat’ basis. Participants’ data will be analysed based on the group to which they have been assigned, irrespective of attrition. IBM SPSS Statistics 27 software will be used to analyse the data.

A CONSORT diagram will outline the number of participants who were identified, recruited, commenced and finished the intervention, with recruitment rate reported as a percentage. Reasons for refusal and dropout during the intervention will also be recorded and reported. Descriptive statistics will be used to present baseline characteristics and feasibility outcomes. Inferential statistics (correlations and analysis of variance — ANCOVA) will be used as indicators of difference between the intervention and control arms and correlation between baseline and follow-up measurements. We will use data from this study, together with that from previous research, to inform a suitable sample size for a future effectiveness trial.

## Discussion

Interventions for family carers with a training and support component have been shown to be effective in reducing levels of stress and improving quality of life [7–9]. However, these interventions are often delivered face to face, which can be a barrier to some family carers’ participation [10]. The online delivery of the current intervention offers flexibility, removing these barriers [11, 12] and encouraging and promoting participation. Despite the potential benefits of the Carers-ID intervention, its feasibility is unknown.

The Carers-ID intervention was co-designed with family carers of people with ID and voluntary sector partners and is therefore directly relevant to their lives. Our initial testing has shown that the programme is acceptable to family carers who felt that providing peer support and the opportunity to talk to other carers was an important element of the work. However, our use of convenience sampling in the absence of a sampling frame from which to draw a list of eligible participants may mean that our sample lacks representativeness to the wider population of family carers of people with ID. We intend to address this issue by recruiting from across the four countries of the UK and providing all who are interested in taking part with the opportunity to do so.

The mixed-methods design in the current study will provide important information on the feasibility of the Carers-ID online intervention. It will provide us with useful data on how our chosen outcome measures perform while also contributing information on the mental health of family carers. Findings from this trial will inform the appropriateness of progression to a phase III effectiveness trial, in addition to offering further recommendations to the content of the intervention, if required.

## Data Availability

The authors confirm that data sharing is not applicable to this article as no new data were created or analysed in this manuscript.

## Abbreviations

CONSORT: Consolidated Standards of Reporting Trials
DASS-21: Depression, Anxiety and Stress Scale-21
ID: Intellectual disabilities
MRC: Medical Research Council
SCS-R: Social Connectedness Scale Revised
UK: United Kingdom
WEMWBS: Warwick–Edinburgh Mental Well-being Scale
